# Secondary prevention for intracranial atherosclerotic stenosis: Where we stand and challenges ahead

**DOI:** 10.1515/jtim-2024-0037

**Published:** 2025-01-10

**Authors:** Wanwan Zhang, Erlan Yu, Wenbo Zhao, Chuanjie Wu, Xunming Ji

**Affiliations:** Department of Neurology, Xuanwu Hospital, Capital Medical University, Beijing 100053, China; Department of Neurosurgery, Xuanwu Hospital, Capital Medical University, Beijing 100053, China

## Introduction

**Figure 1:**
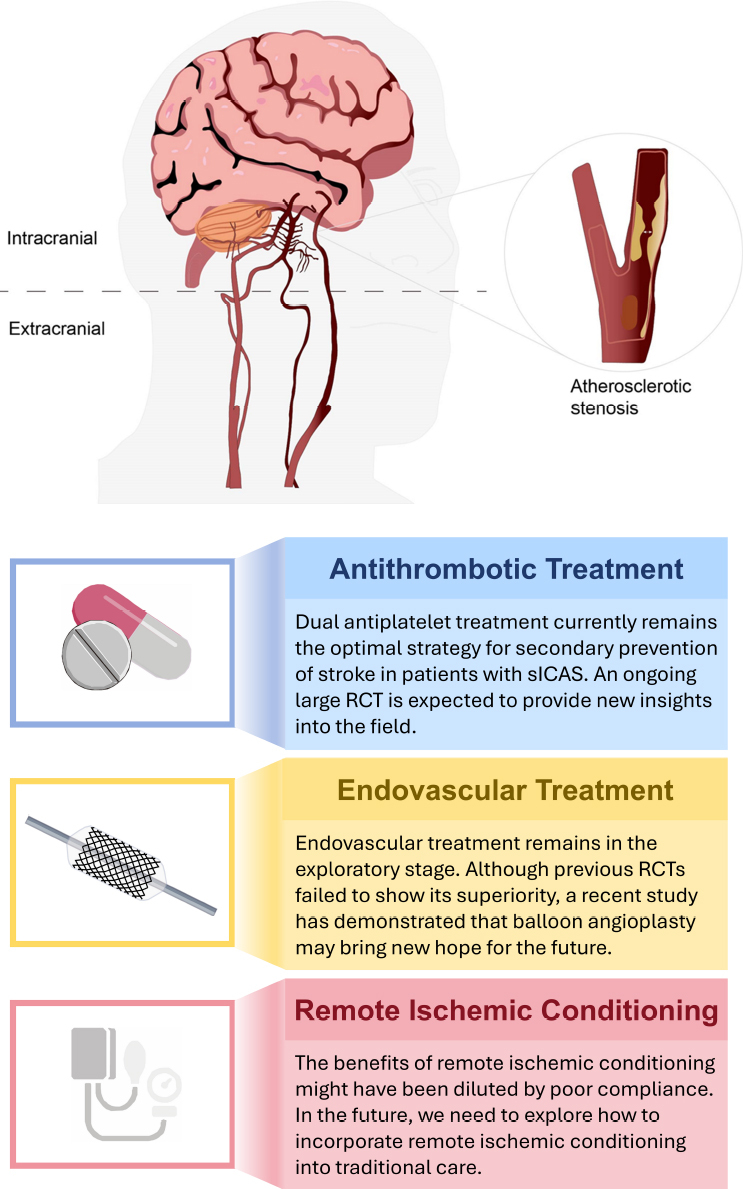
Conclusions of major randomized controlled trials studying secondary stroke prevention in patients with symptomatic intracranial atherosclerotic stenosis. sICAS: symptomatic intracranial atherosclerotic stenosis; RCT: random controlled trials.

Symptomatic intracranial atherosclerotic stenosis (sICAS), usually defined as a recent ischemic stroke or transient ischemic attack (TIA) attributed to 50% to 99% atherosclerotic stenosis of a relevant intracranial artery. It is a major etiology of stroke worldwide, and is associated with a high risk of recurrent stroke, especially in East and South Asia. There is an approximately 15% risk of the 1-year recurrent stroke in patients with sICAS. Current treatment strategies for sICAS primarily include medical management, surgical intervention, and endovascular treatment (EVT). Among these, surgical treatment has not been widely adopted in clinical practice due to its relatively high recurrence rate. The optimal prioritization between EVT and medical management for sICAS is still under investigation. Current evidence continues to favor medical management as the first-line treatment for ICAS patients. Recently, however, the Balloon Angioplasty for Symptomatic Intracranial Artery Stenosis trial (BASIS trial; *n* = 501), investigating balloon angioplasty plus aggressive medical management in patients with severe sICAS (70% to 99% atherosclerotic stenosis of a relevant intracranial artery), found that EVT outperforms aggressive medical management.^[1]^ It may reignite the research prospects for EVT in the sICAS field after its setbacks. Hence, this review outlines the current and emerging major random controlled trials (RCT) in sICAS patients, and highlights promising future therapeutic strategies (Figure 1).

## Antithrombotic treatment

Since the Warfarin Aspirin Symptomatic Intracranial Disease trial (WASID trial; *n* = 569) published in 2005, antithrombotic drugs, represented by aspirin, have gradually become the preferred regimen for secondary prevention of stroke in sICAS.^[2]^ Subsequently, several large RCTs successively demonstrated that aspirin combined with clopidogrel significantly reduced the risk of stroke recurrence, compared with aspirin alone.^[3,4]^ Therefore, an increasing number of international guidelines recommended dual antiplatelet therapy as the standard treatment strategy for sICAS. However, further studies have shown that some patients carrying CYP2C19 loss-of-function alleles (25% of White patients and 60% of Asian patients) may not benefit from clopidogrel treatment. For these patients, ticagrelor or cilostazol may be considered as alternative options, but their efficacy and safety still require further validation. The Comparison of Anti-Coagulation and Anti-Platelet Therapies for Intracranial Vascular Atherostenosis trial (CAPTIVA trial; NCT05047172) is an ongoing large-scale RCT designed to comparing the efficacy of three drugs combinations—ticagrelor and aspirin, low-dose rivaroxaban and aspirin, and clopidogrel and aspirin—in patients with sICAS. With the aim of determining the optimal antithrombotic combinations, the trial is estimated to enroll 1683 patients and be completed by 2029, providing stronger evidence-based support for the selection of treatment regiments for sICAS patients.

## EVT

Although pharmacological treatment remains the first-line treatment for sICAS, its effect of stroke prevention is not ideal for some patients. For over a decade, whether EVT is superior to medical therapy alone for sICAS has been a controversial and unresolved issue. The results of the early Stenting and Aggressive Medical Management for Preventing Recurrent Stroke in Intracranial Stenosis (SAMMPRIS; *n* = 451) and Vitesse Intracranial Stent Study for Ischemic Stroke Therapy (VISSIT; *n* = 112) trials showed that medical therapy alone was superior to EVT (self-expanding stents and balloon-expandable stents), leading to a decline in researches on EVT for sICAS.^[4,5]^ With in-depth analysis of previous studies, researchers have begun to attempt to establish more stringent patient selection criteria and advanced treatment strategies, aiming to help more sICAS patients benefit from EVT. In 2022, the China Angioplasty and Stenting for Symptomatic Intracranial Severe Stenosis trial (CASSISS trial; *n* = 358) found no significant difference in the risk of stroke or death comparing percutaneous transluminal angioplasty and stenting to medical therapy alone in sICAS patients.^[6]^ The trial primarily focused on low-risk populations, excluding patients with isolated perforator artery infarction and those who had experienced a stroke or TIA within the past three weeks. However, the negative results of CASSISS indicated that our understanding of sICAS is still superficial, and the high-risk populations truly suitable for EVT have not been properly identified. In recent years, new interventional devices, such as drug-coated balloons and biodegradable stents, have continuously emerged in the field of ICAS treatment.^[7]^ The NOVA trial (Comparison of Drug-Eluting Stent With Bare-Metal Stent in Patients With Symptomatic High-Grade Intracranial Atherosclerotic Stenosis; *n* = 263) demonstrated that new drug-eluting stents significantly outperformed bare-metal stents for sICAS, and reduce the risk of stroke recurrence and in-stent restenosis.^[8]^ Encouragingly, an increasing number of studies suggested that for specific sICAS patients, the potential benefits of EVT are likely to be further confirmed. The BASIS trial published recently, confirmed that balloon angioplasty plus aggressive medical management significantly reduced the risk of stroke recurrence and death. This is the first RCT in the field of EVT for sICAS to yield positive results, offering a new treatment option for these patients.^[1]^ Compared to previous studies, the BASIS trial included patients with a recent TIA (< 90 days) or ischemic stroke (14–90 days), and employed submaximal balloon angioplasty without stent implantation. The success of this trial may be attributed to some factors such as appropriate timing of intervention, high-risk patient populations, and safer techniques of EVT. Therefore, how to accurately identify patients with drug-refractory sICAS, grasp the optimal timing of intervention, and optimize treatment strategies to reduce the risk of periprocedural complications and prevent stroke recurrence are critical issues that need to be addressed and will be the focus of future research in this field.

## Remote ischemic conditioning (RIC) trials

RIC is a promising treatment in sICAS, which protects the brain from subsequent ischemic injury by repetitive transient ischemia of limbs.^[9]^ At present, the largest RCT to enroll sICAS patients was the RICA trial (Chronic remote ischaemic conditioning in patients with sICAS; *n* = 3033), which aimed to evaluate the effect of RIC for stroke prevention.^[10]^ RICA was a sham-controlled trial at 84 stroke centers in China, including patients within 30 days of ischemic stroke or TIA. And the RIC device was placed on the both upper arms of the patients lasting for 45 min daily over one year. Regrettably, there was no significant difference in ischemic stroke incidence between the two study groups due to poor compliance (hazard ratio 0.87, 95% confidence interval (CI) 0.74–1.03, *P* = 0.12). In the future, we should initiate RIC early in the targeted population, and improve patient compliance to further verify the effect of RIC.

## Conclusion

In summary, although some important advances in the treatment of sICAS over the past 20 years, the risk of stroke recurrence is still high. Prioritized research areas for sICAS include exploring the optimal antithrombotic combinations, refining sICAS patient selection, improving treatment strategies, and increasing adherence to risk factor control. Despite there are numerous research opportunities in the future, sICAS therapeutic trials must focused on the population with high risk of recurrent stroke who are the most in need of these innovative treatments. We hope that this review will stimulate further studies to discover more effective secondary prevention treatments in sICAS.
